# Professionalism among multicultural medical students in the United Arab Emirates

**DOI:** 10.1080/10872981.2017.1372669

**Published:** 2017-09-18

**Authors:** Mahera Abdulrahman, Shahd Alsalehi, Zahra S. M. Husain, Satish C. Nair, Frederick Robert Carrick

**Affiliations:** ^a^ Department of Medical Education, Dubai Health Authority, Dubai, United Arab Emirates; ^b^ Department of Primary Health Care, Dubai Medical College, Dubai, United Arab Emirates; ^c^ Department of Academic Affairs, Tawam Hospital, College of Medicine, UAE University, Al Ain, United Arab Emirates; ^d^ Bedfordshire Centre for Mental Health Research, University of Cambridge, Cambridge, UK; ^e^ Neurology, Carrick Institute, Cape Canaveral, FL, USA; ^f^ Harvard Macy Institutes, Boston, MA, USA; ^g^ MGH Institutes, Boston, MA, USA

**Keywords:** Professionalism, academic misconduct, medical students, United Arab Emirates

## Abstract

**Background**: Moral competencies and ethical practices of medical professionals are among the desired outcomes of academic training. Unfortunately, academic dishonesty and misconduct are reported from medical colleges across the world. This study investigates the level of academic dishonesty/misconduct among multicultural medical students.

**Objective:** The aim of this study is to investigate the level of academic dishonesty/misconduct among multicultural medical students.

**Design**: Validated and customized version of Dundee Polyprofessionalism Inventory-1 detailing lapses of professionalism in undergraduate health professions education was used to determine the perceived prevalence and self-reported lapses of academic integrity in this study.

**Results**: This study shows that the majority (458/554, 83%) of medical students have admitted to acts of academic dishonesty mentioned in the questionnaire. Approximately 42% (231/554) of the students have given proxy for attendance and 71% of them considered this as an offense. Similarly, 12% (66/554) have copied from the record books of others, and 86% (477/554) have considered it unethical. In addition, 5% (28/554) of the students revealed forging a teacher’s signature in their record or logbooks, with 16% (91/554) of them reporting that they have seen others forge signatures.

**Conclusion**: This is the first multi-center, multi-cultural and multi-ethnic study involving a large number of participants that addresses academic professionalism among medical students in the Middle East. Certainly, the paucity of data limits definitive conclusions about the best approach to prevent academic misconduct in the UAE medical schools. Yet, the results of our study are anticipated not only to benefit the UAE but also to find application in the Arab world, with similar medical school programs, values, culture and tradition.

## Introduction

The pressures of performance and productivity may affect professionalism. Self-interest stemming from commercial, financial, and political pressures has strained medical professionalism, reducing the public respect towards physicians and physician-scientists. Moral competencies and ethical practices of medical professionals are desired outcomes of academic training.[1] Yet, academic dishonesty/misconduct such as cheating, plagiarism, falsifying and fabricating documents, is common in medical colleges across the world, and it seems that its incidence is on rise.[2] There is growing evidence that academic dishonesty is widespread in medical and health care schools worldwide.[3] These actions have a detrimental effect on medical practice because students who cheat during medical school appear to follow the same behavioral pattern later on in their work with patients.[3] Such students lack the required competencies to become safe doctors and may not be considered fit to practice. Recently, critics and observers of the medical fraternity have urged that more attention needs to be invested in the moral and ethical qualities of medical students. Transmitting moral values and ethical practices from senior physicians to juniors has always been an essential core of medical training. Therefore, the urge to reform medical training towards moral sensibilities is warranted.

Worldwide, there is wide variation in literature reviews of the numbers of students self-reporting academic misconduct in medical schools. The variation is between 2% [2] in the UK and 99% [4] in Croatia. A multiregional study involving medical practitioners from UK, Europe, North America, and Asia suggested that the variation in aspects of professionalism might be due to diverse cultural and socio-economic backgrounds.[5]

United Arab Emirates (UAE) is a fast growing country with more than 180 nationalities. Although a Middle Eastern culture and Islamic values are predominant in UAE, Western and Far Eastern beliefs and standards can be widely noticed in the community. Nevertheless, having students from different nationalities [6–8] and cultural backgrounds makes the appraisal of academic dishonesty even more challenging in UAE. The primary purpose of this study is to assess the prevalence of the academic misconduct among the medical students in UAE medical schools. The secondary purpose is to determine whether gender, nationality, upbringing, and year of study of the students contribute to misconduct.

## Subjects and methods

### Study design and setting

The study design was cross sectional and carried out at three undergraduate medical schools in UAE: Dubai Medical College, Ras Al Khaimah Medical and Health Sciences University, and Gulf Medical University. Surveys were conducted from September to December 2016. Medical students were approached and questionnaires were distributed in the classrooms at the end of lectures. Students were briefed about the study and were asked to voluntarily participate in the study after consent. Students were assured that they could withdraw at any time without reprisal and that their anonymity will be maintained. Aggregate reporting of data was assured to enhance confidentiality and accurate reporting by the respondents. Anonymity of participation was also guaranteed by return of completed survey constructs to an administrator; independent and blinded to the study hypothesis. A code linking respondents to their surveys was kept isolated from the investigators.

### Evaluation tools

The survey used in this study consisted of two major components: Socio-demographic characteristics, and The Dundee Polyprofessionalism Inventory-1.

#### Socio-demographic characteristics questionnaire

Demographic data including gender, age, nationality, place of upbringing, location of medical school, and year of study were recorded.

#### The Dundee Polyprofessionalism Inventory-1

Validated and customized version of Dundee Polyprofessionalism Inventory-1 [9] consisting of 40 general items outlining lapses of professionalism in undergraduate health professions education was used to determine the perceived prevalence and self-reported lapses of academic integrity in this study. Participants had to choose between the options: yes, no, or unsure for 20 questions assessing behavior. Each question on behavior was further divided into two parts: (a) the student is wrong and (b) have done or would consider doing the same, to yield a total of 40 responses.

### Data analysis and statistics

Descriptive statistics were computed for the socio-demographic variables, and professionalism perceptions. The frequency of the responses for each behavior (option: yes) was expressed as percentage of total, in addition to actual number of responses. The significance in the difference for prevalence between the subgroups such as male and female (gender analysis) was determined using the chi square test 95% CI (SPSS Version 23, IBM; (IBM Corp., Released 2011, Armonk, NY, USA)). Independent t-tests and analyses of variance (ANOVAs) were performed to analyze the differences between demographic variables and academic misconduct.

### Ethics statement

The study was approved by the Research Ethics Committee of Dubai Medical College, Dubai. Participants were not compensated. All participants gave written informed consent before participation.

## Results

The College of Medicine program between the three medical colleges surveyed for this study, Dubai Medical College, RAK Medical and Health Sciences University, and Gulf Medical University has approximately 1300 medical students enrolled, from the first year to final year medical degree program. 910 students were approached with 554 medical students providing consent to participate, yielding an overall response rate of 61%. Our study has attempted to categorize and analyze the responses provided by the participants based on gender, nationality, upbringing and years of study (training). The distribution of students at all levels of medical school training was almost even. A large number, 39% (214/554), of the students were from DMC. The distribution of age groups was skewed towards a lower age group (less than 22 years, 84%) and the participants were predominantly females (435, 78%). The UAE nationals contributed to 11% of the total participants compared to non-nationals; however, the majority (354, 64%) of the medical student participants were raised in the UAE.

### Prevalence of academic misconduct

The majority (458/554, 83%) of the students reported committing at least one of the acts of academic dishonesty mentioned in the questionnaire. About 42% (231/554) of the students have given proxy for attendance and 71% of them considered it as an offense. Similarly, 12% (66/554) of them have copied from the record books of others, with 86% (477/554) considering this practice to be unethical. Interestingly, 5% (28/554) of the students admitted to forging a teacher’s signature in their record or logbooks, but 16% (91/554) reported having seen others doing it (Table 1). Astoundingly, approximately 23% (126/554) of the students tried to obtain examination questions before the commencement of the exam. Twelve percent (66/554) reported that they copied from their friends’ answer sheet, 6% (33/554) reported that they copied from unauthorized study material during their exams, and 4% (23/554) have tried to change grades in the official record.

**Table 1.** Prevalence of academic misconduct among students in UAE`s medical schools.

Category	(n)	%
Students have done at least one of the acts of academic dishonesty mentioned in the questionnaire	458/554	83
Students have given proxy for attendance	231/554	42
Students considered giving attendance as an offense	393/554	71
Students have copied from others record book	66/554	12
Students considered coping from others record book unethical	477/554	86
Students revealed forging teacher’s signature in their record or logbooks	28/554	5
Students have seen others forging teacher’s signature in their record or logbooks	91/554	16
Students tried to get the exam question paper before the commencement of the exam	126/554	23
Students have copied from their friends’ answer sheet	66/554	12
Students have copied from unauthorized study material during their exams	33/554	6
Students have tried to change grades in the official record	23/554	4

### Gender differences

Striking gender differences were observed in the responses. More than two-thirds (68%) of the female students affirmed that ‘lack of punctuality’ was wrong compared to 17% of their male counterparts (*p *< 0.001). Similarly, significantly higher percentages of female students compared to male students perceived and accepted being ‘wrong’ for the items related to: creating unrelated circumstances to delay sitting for an exam (female 70% vs. male 16%, *p *< 0.001), furbishing someone else’s intellectual property as one’s own (female 75% vs. male 16%, *p *< 0.001), copying answers from a neighbor (female 73% vs. male 16%, *p *< 0.001), claiming team work as individual effort (female 48% vs. male 9%, *p *< 0.05), substituting for examinations (female 74% vs. male 18%, *p *< 0.001), and fabricating to cover up mistakes in records (female 79% vs. male (17%, *p *< 0.001).

Differences in gender responses were also noted for the items related to: signing attendance sheets in absentia (female 59% vs. male 13%, *p *< 0.05), missing lectures frequently (female 65% vs. male 15%, *p *< 0.05), falsifying bio sketches (female 69% vs. male 17%, *p *< 0.05) and changing grades in official records (female 73% vs. male 18%, *p *< 0.05). Overall, the responses to the question ‘having done wrong and would consider doing it again’ were scanty and restricted to 2–35%. Female students (6%) were repeatedly more prone to ‘making false entries in their logbooks’ (6%, *p *< 0.001) and ‘presenting someone else’s work as one’s own’ (6%, *p *< 0.001) as compared to their male counterparts. Female students were more likely to ‘take cheat sheets to the exam’, ‘claiming teamwork as one’s individual effort’, and ‘signing attendance sheet for others’ (*p *< 0.001).

### Nationality differences

Being a UAE national or an expatriate was found to be an important variable in assessing professionalism in the UAE. Items measuring professionalism related to ‘lack of punctuality for classes (non-national 75% vs. national 10%, *p *< 0.001)’, ‘missing lectures’ (non-national 70% vs. national 9%, *p *< 0.001), ‘presenting or signing false certificates’ (non-national 82% vs. national 11%, *p *< 0.001), ‘taking work idea from others and presenting it as one’s own’ (non-national 80% vs. national 10%, *p *< 0.001) were significantly accepted as ‘wrong’ by the non-national (expatriates) as compared to the nationals (citizens) of the UAE. Statistical differences were also observed in the responses between the UAE national medical students for the items: ‘signing attendance sheet for others’ (non-national 63% vs. national 9%, *p *< 0.05), ‘making false entries in logbook’ (non-national 78% vs. national 10%, *p *< 0.05), ‘inventing unrelated circumstances to delay sitting in an exam’ (non-national 76% vs. national 10%, *p *< 0.05), ‘copying or facilitating copying’ (non-national 79% vs. national 10%, *p *< 0.05), ‘presenting team work as one’s individual effort’ (non-national 78% vs. national 10%, *p *< 0.05), ‘getting or giving help against teachers advice’ (non-national 80% vs. national 10%, *p *< 0.05), ‘taking cheat sheets for exam’ (non-national 82% vs. national 10%, p < 0.05), ‘substituting for exams’ (non-national 81% vs. national 10%, *p *< 0.05),’ and ‘fabricating records to cover mistakes’ (non-national 81% vs. national 10%, *p *< 0.05). The non-UAE students responded differently to the question ‘having done wrong and would consider doing it again’ as compared to the UAE nationals. The UAE national medical students were less likely to ‘fabricate test results to cover their mistakes’ and ‘claim teamwork as individual effort’, *p *< 0.05), compared to non-nationals.

### Upbringing differences

Almost half (55%) of the medical students raised (upbringing) in the UAE responded that ‘lack of punctuality’ was wrong compared to 29% of their non-UAE raised counterparts. The differences between the two groups were highly significant (*p *< 0.001). Interestingly, except for ‘missing lectures’ where differences in responses were noted (*p *< 0.05) between the groups in terms of upbringing, no other differences were observed. The variable ‘whether the medical students were raised in the UAE or elsewhere’ had little impact on the question ‘having done wrong and would consider doing it again.’ The non-national medical students were more likely to engage in ‘receiving information about an examination from a student who have already taken the exam’ compared to the nationals (*p *< 0.001). In contrast, the UAE nationals were more prone to ‘complete work for another student’ and ‘not doing the assigned part in their group’ (*p *< 0.05).

### Academic year differences

Data presented was categorized into two groups: (a) preclinical (years 1 and 2) and (b) clinical (years 3–5). Irrespective of academic year of study, both the preclinical and the clinical groups acknowledged ‘lack of punctuality’ as an academic misconduct. However, repetitive behavior was more prevalent among the students in their later years of study (*p* < 0.001, Table 2), compared to their early counterparts. The students in the clinical years significantly differed (*p *< 0.001) in their acknowledgement of the fact that ‘claiming team work as individual effort was wrong’ (Table 2). Overall, the responses did not vary significantly between the pre-clinical and the clinical groups for all the other items on the survey.

**Table 2.** Analyze of the the responses provided by the medical students based on gender, nationality, upbringing and years of study (training).

		*Gender*		*Nationality*		*Upbringing*		*Academic year*	
		*Female*	*Male*	*UAE*	*Non UAE*	*UAE*	*Non UAE*	*Pre-clinical (Years 1-2)*	*Clinical (Years 3-5)*
		n (%)	n (%)	n (%)	n (%)	n (%)	n (%)	n (%)	n (%)
1	Lack of Punctuality/timekeeping for classes								
	a. The student is wrong	374 (68)	91 (17)***	55 (10)	409 (75)***	302(55)	161 (29)***	227 (41)	238 (43)
	b. Have done or would consider doing the same	178 (33)	54 (10)	24(4)	206 (38)	147(27)	81 (15)	86 (16)	146 (27)***
2	Signing attendance sheet for absent friends, or asking classmates to sign attendance sheets for you in labs or lectures								
	a. The student is wrong	322 (59)	71 (13)*	47 (9)	346 (63)*	258 (47)	132 (24)	192 (35)	200 (36)
	b. Have done or would consider doing the same	178 (33)	53 (10)***	28 (5)	201 (37)	156 (29)	71 (13)	97 (18)	134 (24)
3	Missing lectures frequently								
	a. The student is wrong	356 (65)	81 (15)*	51 (9)	385 (70)***	290 (53)	144 (26)*	221 (40)	45 (8)
	b. Have done or would consider doing the same	107 (20)	32 (6)	20 (4)	117 (21)	91 (17)	47 (9)	45 (8)	14 (2.6)***
4	Falsifying references or grades on curriculum vitae								
	a. The student is wrong	377 (69)	93 (17)*	55 (10)	413 (76)	306 (56)	160 (29)	227 (42)	243 (44)
	b. Have done or would consider doing the same	26 (5)	14 (3)	9 (2)	31 (6)	27 (5)	12 (2)	10 (2)	29 (5)
5	Changing grades in official record								
	a. The student is wrong	400 (73)	99 (18)*	56 (10)	440 (80)	324 (59)	170 (31)	246 (45)	252 (46)
	b. Have done or would consider doing the same	12 (2)	11 (2)	4 (1)	19 (3)	15 (3)	7 (1)	8 (1)	15 (3)
6	Presenting false certificates/signing false certificates								
	a. The student is wrong	401 (73)	106 (19)	58 (11)	448 (82)***	330 (60)	172 (31)	251 (46)	255 (46)
	b. Have done or would consider doing the same	11 (2)	16 (2)	4 (1)	22 (4)	16 (3)	10 (2)	6 (1)	21 (4)
7	Making false entries in logbook / signing such logbooks								
	a. The student is wrong	394 (72)	91 (17)*	56 (10)	429 (78)*	317 (58)	164 (30)	239 (44)	247 (45)
	b. Have done or would consider doing the same	34 (6)	18 (3)***	7 (44)	44 (8)	35 (6)	17 (3)	14 (3)	38 (7)
8	Inventing unrelated or irrelevant circumstances to delay sitting in an exam								
	a. The student is wrong	282 (70)	87 (16)***	53 (10)	415 (76)*	305 (56)	161 (29)	229 (42)	239 (44)
	b. Have done or would consider doing the same	23 (4)	14 (3)*	5 (1)	21 (4)	23 (4)	14 (3)	14 (3)	23 (4)
9	Take the work or idea from a fellow student and passing it off as one’s own without acknowledging it or purchasing work from a supplier								
	a. The student is wrong	409 (75)	88 (16)***	57 (10)	439 (80)***	321 (59)	172 (32)	234 (43)	253 (46)
	b. Have done or would consider doing the same	32 (6)	20 (4)***	7 (1)	44 (8)	30 (6)	21 (4)	20 (4)	32 (6)
10	Not doing the part assigned to him/her In group work								
	a. The student is wrong	400 (73)	96 (18)*	5 7(10)	438 (80)	324 (59)	168 (31)	242 (44)	253 (46)
	b. Have done or would consider doing the same	18 (3)	16 (3)	5 (1)	28 (5)	19 (13)	14 (3)*	12 (2)	22 (4)
11	Completing work for another student								
	a. The student is wrong	231 (42)	61 (11)	30 (6)	261 (48)	179 (33)	112 (21)	146 (27)	146 (27)
	b. Have done or would consider doing the same	185 (34)	34 (7)	27 (5)	175 (32)	142 (26)	57 (10)*	89 (16)	113 (21)
12	Accessing old exam papers or coursework, which have not been released to the whole class to assist in study								
	a. The student is wrong	275 (51)	63 (12)	40 (7)	297 (55)	215 (40)	120 (22)	160 (29)	178 (32)
	b. Have done or would consider doing the same	137 (25)	25 (8)	24 (4)	155 (28)	121 (22)	57 (10)	74 (14)	105 (19)
13	Copying answers from a neighbor or enabling a neighbor to copy your answers during an exam								
	a. The student is wrong	398 (73)	90 (16)***	57 (10)	430 (79)*	315 (58)	168 (31)	245 (45)	242 (44)
	b. Have done or would consider doing the same	28 (5)	10 (2)*	6 (1)	31 (6)	23 (4)	14 (3)	12 (2)	26 (5)
14	Exchanging answers using mobile phones during an exam								
	a. The student is wrong	225 (42)	61 (11)	35 (6)	250 (46)	176 (33)	106 (20)	139 (26)	147 (27)
	b. Have done or would consider doing the same	99 (18)	27 (5)	18 (3)	107 (20)	85 (16)	40 (7)	52 → 10	74 → 13.6
15	Claiming Team work as one’s individual effort								
	a. The student is wrong	391 (48)	72 (9)***	53 (10)	423 (78)*	311 (57)	162 (30)	232 (42)	245 (45)***
	b. Have done or would consider doing the same	86 (16)	18 (3)***	11 (2)	54 (10)*	38 (7)	27 (5)	30 (6)	36 (7)
16	Getting or giving help for coursework, against a teacher’s advice (e.g., lending work to another student to look at)								
	a. The student is wrong	399 (73)	82 (15)***	54 (10)	436 (80)*	317 (58)	171 (31)	241 (44)	249 (45)
	b. Have done or would consider doing the same	20 (4)	11 (2)***	6 (1)	24 (4)	17 (3)	12 (2)	11 (2)	20 (4)
17	Receiving information about the paper from a student who have already sat in the exam, or providing information about a paper to students who have yet to sit in it								
	a. The student is wrong	301 (55)	75 (14)	44 (8)	330 (60)	238 (44)	134 (25)	185 (34)	190 (35)
	b. Have done or would consider doing the same	97 (18)	29 (5)*	15 (3)	110 (20)	89 (16)	234 (43)***	47 (9)	78 (14)
18	Taking unauthorized materials (e.g., cheat sheets, ‘‘Bootee’’) into an exam								
	a. The student is wrong	408 (75)	98 (18)*	57 (10)	447 (82)*	329 (60)	173 (32)	245 (45)	260 (48)
	b. Have done or would consider doing the same	23 (4)	10 (2)***	8 (1)	24 (4)	17 (3)	16 (3)	11 (2)	22 (4)
19	Sitting an examination for someone else, or someone else sit an examination for you								
	a. The student is wrong	402 (74)	99 (18)***	56 (10)	439 (81)*	321 (59)	171 (31)	242 (44)	253 (46)
	b. Have done or would consider doing the same	18 (3)	10 (2)	3 (1)	25 (5)	19 (3)	9 (2)	9 (2)	19 (3.5)
20	Intentionally fabricating the test results or treatment records in order to cover mistakes								
	a. The student is wrong	404 (79)	91 (17)***	55 (10)	439 (81)*	320 (59)	171 (32)	239 (44)	255 (47)
	b. Have done or would consider doing the same	8 (2)	14 (3)	5 (1)	19 (4)*	14 (3)	11 (2)	7 (1)	17 (3)

***p<0.001, *p < 0.05, significance determined using Montecarlo 2 tailed significance at 95% CI

## Discussion

Although medical professionalism relates to a set of attributes and behaviors, the context of it may change when applied to a multicultural environment such as the United Arab Emirates. Moreover, professionalism cannot be taken out of a social context involving the healers and the healed.[10] Evidence has continually suggested that unethical behavior while in school can translate to unethical, and even criminal, behavior in the workplace.[11] Thus, it is of paramount importance to attempt to better understand the phenomena of academic misconduct and to utilize this knowledge in the reduction of such behaviors, especially in a rapidly growing country like the UAE.

Recently, almost 42% of freshmen at Harvard University admitted to cheating on a homework assignment during their time in high school.[12] Another study raised an alarm when 81.7% of college alumni admitted to academic misconduct during their college years in the USA.[13] The present study also indicates that almost all of the medical student participants in the UAE were involved in at least one act of academic dishonesty, the most common ones being giving the proxy for attendance, getting technical help during exams and copying during exams. Studies have shown that the rates of academic dishonesty vary among students of different origins. Seventy percent of students from the USA have admitted cheating at some point during their college days, while 64% of Russian and 84% of Polish undergraduates admitted to cheating.[14] In contrast to these high rates of copying among Russian and Polish students, only 2% of British students confessed to copying at their degree examinations.[2] Medical students demonstrating unprofessional behavior in medical school were more likely to have a subsequent state board disciplinary action.[15] Interestingly, the observation that male students and non-nationals were likely to cheat more than their respective counterparts from our study, needs further examination given the relatively low number of participants in these sub-categories (Table 2). The high number of female medical students and high volume of non-nationals are the unique attributes of the medical school program in the UAE.[8]

Medical schools have used a variety of techniques to address concerns with students who cheat, including expulsion, reprimands, counseling, and peer review.[16] It is speculated that the faculty and the administration of UAE medical schools may be reluctant to pursue an expulsion because of a lack of a national consensus to support punitive action against academic misconduct. Certainly, the paucity of data limits definitive conclusions about the best approach to prevent academic misconduct in UAE medical schools. An immediate solution to address academic misconduct in the UAE may be to teach moral principles and ethical foundations as the core values of medicine using cases drawn from the local environment.

### Limitations and strengths

To our knowledge, this is the largest study that addresses academic professionalism among medical students in the Middle East. Our study is important given the high rate of burnout experienced by participants in the medical training program in the UAE. The strength of the study is in the generalization that can be obtained from a multi-center, multi-cultural and multi-ethnic study involving a large number of participants. Further, gender, nationality, year of study, and upbringing of the medical student participant contribution to academic dishonesty has also been addressed and analyzed. Causality or correlation for academic dishonesty is a limitation which has not been ascertained in this study. Although speculative at this time, high burnout may be factors in the high prevalence of academic misconduct in the UAE, and needs further research. The results of our study are anticipated not only to benefit the UAE but also to find application in the Arab world, where there are similar medical school programs, values, culture and traditions.
